# Adaptation planning and the use of climate change projections in local government in England and Germany

**DOI:** 10.1007/s10113-016-1030-3

**Published:** 2016-07-28

**Authors:** Susanne Lorenz, Suraje Dessai, Piers M. Forster, Jouni Paavola

**Affiliations:** School of Earth and Environment and ESRC Centre for Climate Change Economics and Policy, University of Leeds, Leeds, LS2 9JT UK

**Keywords:** Local government, Climate change adaptation, Planning, Climate change projections, Institutions, Regulation

## Abstract

Planning for adaptation to climate change is often regarded to be a local imperative and considered to be more effective if grounded on a solid evidence base and recognisant of relevant climate projections. Research has already documented some of the challenges of making climate information usable in decision-making but has not yet sufficiently reflected on the role of the wider institutional and regulatory context. This article examines the impact of the external institutional context on the use and usability of climate projections in local government through an analysis of 44 planning and climate change (adaptation) documents and 54 semi-structured interviews with planners in England and Germany conducted between July 2013 and May 2014. We show that there is little demand for climate projections in local adaptation planning in either country due to existing policy, legal and regulatory frameworks. Local government in England has not only experienced a decline in use of climate projections, but also the waning of the climate change adaptation agenda more widely, amidst changes in the planning and regulatory framework and severe budget cuts. In Germany, spatial planning makes substantial use of past and present climate data, but the strictly regulated nature of planning prevents the use of climate projections, due to their inherent uncertainties. Findings from the two countries highlight that if we are to better understand the usability of climate projections, we need to be more aware of the institutional context within which planning decisions are made. Otherwise we run the risk of continuing to provide tools and information that are of limited use within their intended context.

**Electronic supplementary material:**

The online version of this article (doi:10.1007/s10113-016-1030-3) contains supplementary material, which is available to authorized users.

## Introduction

Climate change adaptation is considered a global challenge. At the same time, it is widely recognised that it happens across local, regional, national and international scales (Adger et al. 2005). It is frequently argued that specific actions and adaptation planning will need to be undertaken locally. Local government is thus often considered a key deliverer of anticipatory and planned adaptation (e.g. de Oliveira 2009; Hurlimann and March 2012; Measham et al. 2011). Planned adaptation is ‘the result of a deliberate policy decision, based on an awareness that conditions have changed or are about to change and that action is required to return to, maintain, or achieve a desired state’ (Parry et al. 2007: 869). Forward planning in local government involves the provision of critical public services such as spatial planning, green infrastructure, flood risk management, housing and emergency planning (ASC 2012). It is this forward planning for adaptation to a changing climate that is the focus of this article.

‘Planned adaptation to climate change means the *use of information* about present and future climate change to review suitability of current and planned practices, policies, and infrastructure’ (Füssel 2007a: 268, emphasis added). More often than not such information is based on climate projections. ‘A climate projection is the simulated response of the climate system to a scenario of future emission or concentration of greenhouse gases (GHGs) and aerosol’ (Parry et al. 2007: 872). They differ from climate predictions in that they are based on different socio-economic, technological and population development pathways that can reach far into the future, which may or may not become reality. These scenarios thus introduce greater uncertainty into climate projections than climate predictions contain (Parry et al. 2007: 872). Other common sources of information used for planning, particularly in Germany, are climate function maps. These take into account topography, land use and building coverage and show an area-wide representation of the thermal and dynamic microclimate characterised as different climatopes (Heaphy 2014; Matzarakis et al. 2008).

In the climate change context, where the stakes and the uncertainties in the decision processes are regarded as extraordinarily high (Funtowicz and Ravetz 1993), effective and efficient adaptation planning is considered dependent not only on climate information at appropriate scales but also on extending the notion of the ‘expert’ in the decision-making process (Funtowicz and Ravetz 1993; Füssel 2007a). This process thus calls for a more interactive dialogue between and a greater role for scientists, practitioners, decision-makers and stakeholders (Funtowicz and Ravetz 1993; Füssel 2007a; Gibbons et al. 1994). It is proposed that in the process of converting this knowledge into action, the knowledge systems between the different agents, often largely grouped into decision-makers and scientists (or users and producers of information), differ (McNie 2007). This makes it more challenging to ensure effective use of the knowledge.

This joint working at the interface between climate science and policy and the use of climate information has been explored in a variety of different studies (e.g. Archer 2003; Kiem and Austin 2013; Kirchhoff 2013; Tang and Dessai 2012). Research suggests that the interaction at this boundary between knowledge and action can be more effective if an iterative approach is employed, which focuses on the production of usable climate science (Lemos and Morehouse 2005). Usability is considered to exist ‘within a range in which each use is defined by a perception of usefulness and the actual capacity (e.g. human and financial resources, institutional and organizational support, political opportunity) to use different kinds of information’ (Dilling and Lemos 2011: 681). This perception and capacity are influenced by both contextual factors (formal and informal institutions, competing factors in the decision-making process such as organisational preferences towards other types of information instead of climate information, organisational culture, wider cultural context of information use and availability of alternative action pathways) and intrinsic factors (understanding of the decision context, spatial and temporal scales of information, perceived legitimacy of and trust in scientific information and accessibility of information) (Dilling and Lemos 2011).

The research focusing specifically on climate information usability is not very extensive, and whilst a number of studies have driven the research field forward through empirical work (e.g. Kiem and Austin 2013; Tang and Dessai 2012), it was Lemos et al. (2012) who provided a conceptual model on the ‘climate information usability gap’. Their model clearly distinguishes between useful information (as provided by producers of climate information) and usable information (as required by users of climate information). The model sets out to show that information provided by producers needs to pass through a transition space before it reaches the user (Lemos et al. 2012). If this transition space is characterised by little interplay or interaction between users and producers, the information reaching the user will fit poorly with their needs and contexts resulting in low use and usability (Lemos et al. 2012). However, if the transition space is filled with a range of options to enable interaction between producers and users, better tailored and more usable information will reach the users (Lemos et al. 2012). Such tailoring efforts include value-adding (conversion of data to information), retailing and wholesaling (provision of information at appropriate user-defined scales), and customisation (end-of-process information adjustment) (Lemos et al. 2012). Whilst the concept of providing usable information for decision processes is not unique to the provision of climate information, Lemos et al.’s (2012) model focuses on this particular context, as despite a wide range of useful information on climate change being in existence, this information still often goes unused. Due to the inevitability of needing to make climate adaptation decisions (Moss et al. 2013), however, moving the debate on use and usability of information forward is particularly pertinent to this decision context.

Whilst recent research informed by Lemos et al.’s (2012) conceptual model has tried to better understand how the transition from useful to usable information could be facilitated more effectively through more nuanced information tailoring (Lorenz et al. 2015), we argue that to understand the potential of adopting climate information at the local level, a closer examination of the contextual factors influencing the adoption is also warranted. The influence of contextual factors on adaptation has been considered in previous research (e.g. Dannevig and Aall 2015; Glaas et al. 2010). Yet, these factors are often considered too narrowly within the immediate institutional settings; for example, within municipalities, rural communities, or water companies (Kiem and Austin 2013; Kirchhoff 2013; van Stigt et al. 2015). Furthermore, the findings reported to date have not been sufficiently integrated into the climate information usability literature.

Planning (for adaptation) is considered to be a key tool for progressing action on reducing vulnerability to climate impacts (Hurlimann and March 2012), and Local Authorities (LAs) have substantial power over local planning in terms of both strategic decision-making and land-use management (Measham et al. 2011). Past research has found that reasons for slow progress in local adaptation include those that are internal to Local Authorities (internal institutional context) and those that are external, filtering down from higher levels of government (external institutional context) (Measham et al. 2011). The former includes a lack of technical data, unfamiliarity with such data, a lack of political will, unclear or ill-defined responsibilities, competing priorities and lack of expertise (Amundsen et al. 2010; ASC 2012; Baker et al. 2012; Measham et al. 2011). The latter includes a lack of leadership, guidance and consistency from higher-level governments, restrictive policies, shifting political ideologies and a lack of regulation and/or funding (Amundsen et al. 2010; Baker et al. 2012; Hurlimann and March 2012; Lehmann et al. 2015; Naess et al. 2005; Nalau et al. 2015; Porter et al. 2015).

Given the findings in these recent studies, it is the aim of this article to incorporate the previously identified contextual challenges for adaptation planning into Lemos et al.’s (2012) conceptual model on climate information usability. In doing so, the article will achieve a firmer grounding of the discussions on the usability of climate information within the wider field of adaptation planning. In the “Case studies and methods” section, we outline our case studies and methodology. The differences in the integration of climate information in adaptation planning in England and Germany and how these are impacted by the wider contextual setting are described in the “Results and discussion” section. The “Conclusion” section summarises the findings and highlights the contributions to the conceptual model of usability.

## Case studies and methods

This article adopts a case study approach to obtain a better understanding of the factors influencing the potential for use of climate information (Baxter and Jack 2008). A purposive sampling approach (Hay 2010; Onwuegbuzie and Leech 2010) was used to choose Germany and England as case studies. In-depth interviews and planning and other strategic documents formed two complementary layers of material. The use of these two layers enabled us to underpin interview findings with results from the analysis of publicly available documents.

### Case study description and adaptation policy context

**Fig. 1:**
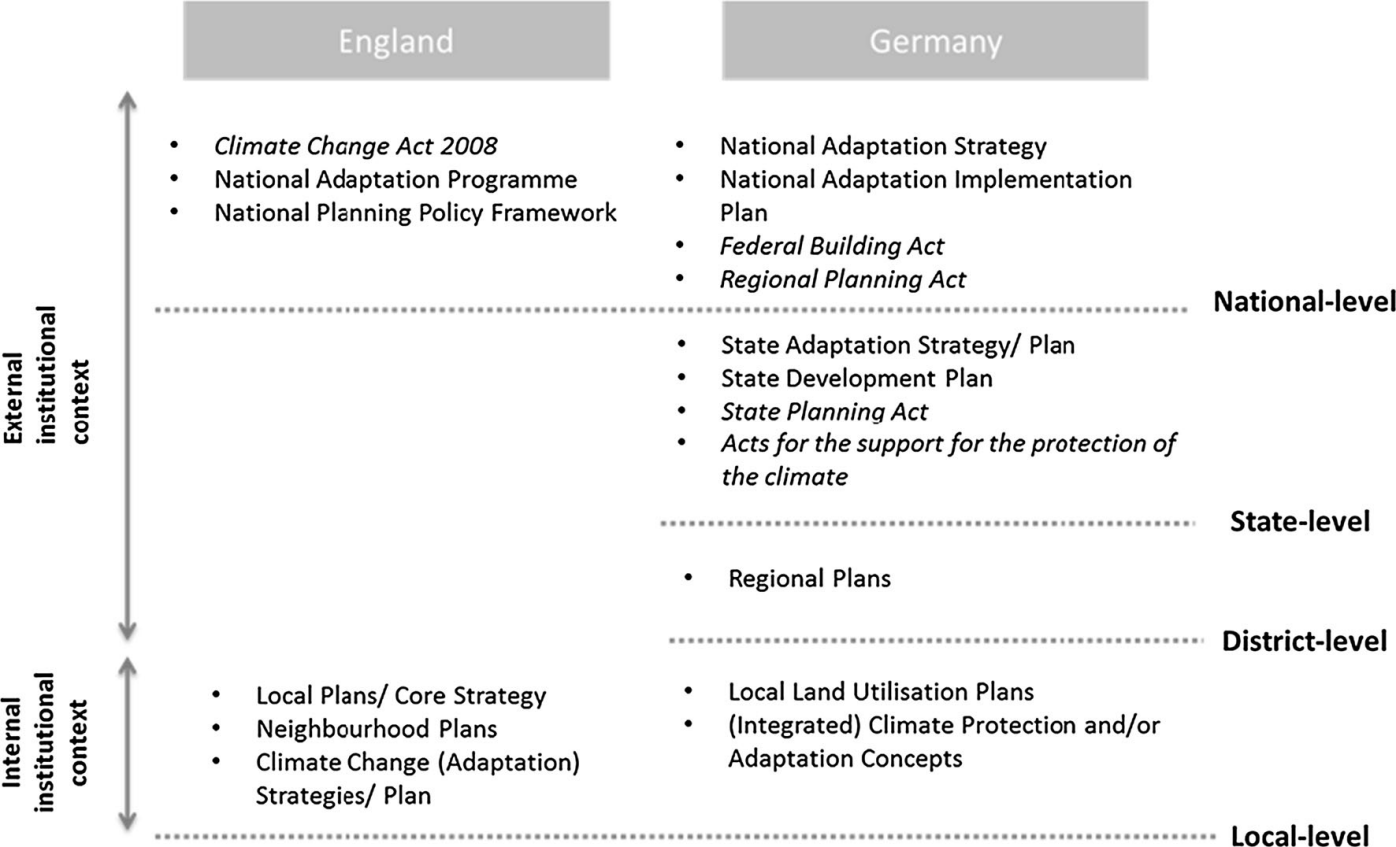
An overview of the legal and policy context of local adaptation planning in England and Germany (Acts are marked in *italics*)

The UK and Germany are both considered leaders in climate change adaptation (Bauer et al. 2012; Massey et al. 2015), even though it has been argued that the UK has shown greater advances in making adaptation a distinctive policy field than Germany (Massey and Huitema 2016). The approaches to adaptation are somewhat different in the two countries. In the UK, the national government plays a key role in agenda setting and coordination (Massey et al. 2015). As some key national adaptation policy documents such as the National Adaptation Plan are specific to the devolved administrations, our analysis focuses on England. In Germany, the states (Länder) play key roles in setting priorities and developing regulatory frameworks whilst national government is the provider of scientific information and financial support (Massey et al. 2015). These differences highlight that we need to be mindful of the different scales at which the institutional context for adaptation planning can be determined (national level in England and state level in Germany). Figure 1 provides an overview of the multi-level legal and policy context of local adaptation planning in the two countries. This context will be explained and explored in more detail in the remainder of the article.

In both countries, local government is a key implementer of adaptation (Massey et al. 2015) and despite some national differences in governance structures, they are largely similar in how climate protection is addressed (Bulkeley and Kern 2006). In Germany, we collected data from one of the 16 federal states, North Rhine-Westphalia, whilst our data from England come from the South East and the East Midlands regions. For a description of the three regions see Online Resource 1.

#### England

In England, the Climate Change Act 2008 contains the key provisions for action on both climate change mitigation and adaptation (Parliament UK 2008). The national government has responsibility to undertake a comprehensive climate change risk assessment (CCRA) every 5 years, which makes use of the UK Climate Projections 2009 (UKCP09), the nationally funded and principal source for UK climate information (both climate projections and observed past climate data). In 2013, a National Adaptation Programme (NAP) was created for England. It considers local government to ‘play(s) a central role in leading and supporting local places to become more resilient to a range of future risks and to be prepared for the opportunities from a changing climate’ (DEFRA 2013: 96). Prior to the change of government in 2010, local authority performance was measured by the Audit Commission using a set of 198 National Indicators (NIs) (DCLG 2007a). Whilst LAs had to report on all indicators, they could prioritise 35 of them in their Local Area Agreement. Three indicators were specific to climate change, with two focused on mitigation and one on adaptation: the process-based indicator *NI188*—*Planning to adapt to climate change*, which provided guidance and helped to measure progress on adaptation. At least one of the three indicators was prioritised in 97 % of LAs (Cooper and Pearce 2011), and whilst NI188 was only prioritised in 30 % of LAs (Cooper and Pearce 2011), it has nevertheless been considered a strong steering mechanism and driver of action (ASC 2012; Boyd et al. 2011). This is due to it having successfully altered the institutional context in favour of climate change action in those LAs in which it was prioritised.

The regulatory and planning framework underwent substantial changes between 2010 and 2015 because of the decentralisation and localism agenda of the Conservative–Liberal coalition government. LAs were no longer required to report to the central government on their performance and the entire indicator set has been terminated. The new National Planning Policy Framework (NPPF), which sets out planning guidance for England, still requires Local Planning Authorities to ‘adopt proactive strategies to mitigate and adapt to climate change’ in their Local Plans (DCLG 2012: 22), but the earlier more detailed Planning Policy Statements, including specific guidance on climate change (DCLG 2007b), have been withdrawn. At the same time, LAs also experienced a 28 % budget cut (Hastings et al. 2015) and have been amongst the hardest hit by the austerity measures (Hastings et al. 2015; Lowndes and Pratchett 2011).

#### Germany

The German political system and administrative structure is decentralised and polycentric (Beck et al. 2009). The Federal Ministry for the Environment, the most important national-level player (Beck et al. 2009; Hustedt 2013), has together with the federal states (Länder) developed a national adaptation strategy (NAS), which it published in 2008. It sets out the overarching framework and guidance for adaptation at the national level (Beck et al. 2009). The implementation plan of the NAS was published in 2011 and is evaluated by the Federal Environment Agency (Hustedt 2013).

The details on the delivery and implementation of adaptation are determined by the policies and goals of the individual Länder. Baden-Wurttemberg and North Rhine-Westphalia (NRW) have even enshrined action on adaptation within their ‘Act for the support for the protection of the climate’. The NRW Act states that ‘the negative impacts of climate change are to be limited through the development and implementation of sector specific adaptation measures that are attuned to the respective regions’ (MIKNRW 2013: 2). Furthermore, states such as Bavaria, Hesse and NRW have published, or are in the process of developing, state adaptation strategies and plans.

At the national level, climate adaptation is specifically mentioned in the Federal Building Act (BJV 2014: Art. 1.5) and the Regional Planning Act (ROG): the latter stipulates that ‘the spatial requirements of climate protection are to be taken into account, through measures that mitigate climate change as well as through those that serve adaptation’ (BJV 2008: 4, Art. 2.6). The latter provision is also reflected in the NRW State Planning Act (MIKNRW 2005). As planning regulations are very hierarchical in Germany, local planning is supposed to fit in and be compatible with higher-level plans. Therefore, a broad overarching framework for local adaptation planning does exist.

### Methods

#### Interviews

We conducted 54 semi-structured interviews with 67 adaptation practitioners at the local, regional, and national level in Germany and England between July 2013 and May 2014. As we focus on planned adaptation, we follow Lehmann et al. (2015: 80) in defining adaptation practitioners as ‘decision-makers in the field of planned climate adaptation’. The majority of the interviewees (*n* = 52) came from the three focus regions mentioned above (England: South East and East Midlands, Germany: NRW). The remaining interviewees (*n* = 15) were based outside of the three regions to ensure that our findings resonate with the German and English experience outside of our focus regions. Our interviewees included local government officials mostly from environment departments (*n* = 51), officials from regional organisations (*n* = 5), district governments (*n* = 1), regional ministries (*n* = 3), regional authorities (*n* = 3), federal authorities (*n* = 2) and the national weather service (*n* = 2).

The core themes covered in the interview protocol included: progress on adaptation within the organisation; the regulatory and statutory framework for action on adaptation; the communication and inclusion of climate projections in strategic documents; the participants’ use of climate projections; and, the participants’ communication preferences regarding climate projections. The interviews were semi-structured to allow for conversations to progress flexibly to the issues and concerns raised by the interviewee. They were conducted either face-to-face or over the phone and were audio-recorded and later transcribed. Transcribed interviews were analysed using software for qualitative analysis (Bazeley and Jackson 2013). Based on the existing literature, we developed an initial coding system that was also allowed to evolve throughout the data analysis process (Harding 2013).

#### Document analysis

We searched and gathered publicly available strategic planning and climate change documents for the LAs with whom we conducted interviews, in the regions we focused on, to triangulate our findings from the interview material. In particular, we analysed whether the documents referred to or used climate projections. We reviewed 14 documents for England and 30 documents for Germany. The differing number of documents reviewed was based upon what was publicly available. For an overview of the material reviewed, see Table 1. For more details on the material in the three focus regions, see Online Resource 2.

**Table 1:** Overview of reviewed documents

	Climate protection concepts	Integrated climate protection and adaptation concept	Land utilisation plans	Regional plans for the districts in NRW	NRW state development plan
Germany	4	5	6	14	1

	Climate change strategies	Climate change adaptation strategies or concepts	Core strategies
England	6	4	4^a^

^a^Only four of the 10 LAs in the focus regions that are Local Planning Authorities have adopted or draft core strategies available online. In the light of the Planning Inspectorate’s latest progress review (2015), this is symptomatic for all English Local Planning Authorities—38 % of them do not have an adopted Local Plan

## Results and discussion

### England

Our analysis shows that local progress on adaptation has largely been driven through government performance indicators. Without the ‘Planning to adapt to climate change’ indicator (NI188), many LAs would not have taken action on adaptation. Whilst the indicator did not dictate how adaptation was to take place, it provided a successful lever to encourage action. In that sense, the government had managed to put institutional drivers in place that were malleable enough to permit context sensitive adaptation. A number of shortcomings of NI188 have been highlighted, such as the perceived lack of detail provided on how to progress through the different stages of adaptation planning or the fact that the indicator lacked the requirement for ‘hard’ action on the ground in the initial stages of adaptation (Cooper and Pearce 2011).

Despite some of these shortcomings, NI188 gave LAs a much-needed direction of travel and five stages to pass through on the way to a regularly reviewed risk-based action plan (LRPB 2010). The risk-based approach to adaptation in England is particularly evident in level 2 of the indicator, which asks for services to be comprehensively assessed against climate (change) impacts. This led the Department for Environment, Food and Rural Affairs (Defra) and the UK Climate Impacts Programme (UKCIP) to advocate and stress the use of climate projections in LAs. To support this to happen, training on the use of the UK Climate Projections 2009 (UKCP09) was provided to some LA officers, to enable assessors to consider possible future states, likelihoods and consequences of potential impacts. However, many LAs failed to generate sufficient information on current and past vulnerabilities, nor on exposure to impacts, to be able to effectively use climate projections to deduce potential future vulnerabilities.

I think what you ended up with was a lot of councils who really thought that it was very important that they used this thing [UKCP09] but had no idea why…Unless you have already done a bit of understanding about what your vulnerabilities have already been, your current risks and the ways you have already been impacted, then you don’t know how to interrogate that properly necessarily. So many of our councils hadn’t done any of that work yet and… I think were not helped by the fact that Defra and the government office were coming over and going, ‘You need to know about this, you are going to use this, it’s going to solve your problems around adaptation’. (Employee of a Regional Organisation, South East England #1)

Due to the novelty of the adaptation agenda and the lack of awareness of vulnerabilities and exposures, it is questionable whether the LAs would have used climate projections to the same extent as they did had it not been for the top-down push.

The use of climate projections also remained confined to awareness raising in the early stages of adaptation planning, rather than becoming integrated throughout the process. Often the projections were not consulted again after local impacts had been identified, ‘largely because they don’t change very much’ (*Employee of a Local Government, South East England #2*). Although the projections could have been of use in planning, for example, as an additional layer on the geographical information system (GIS), this has rarely been done. When it has been tried, it has predominantly been within the climate change or flood risk management team.

The limited capacity of LAs for adaptation planning is also reflected in how comprehensive risk assessments required under NI188 were conducted. The comprehensive risk assessment was intended to cut across all council services to build capacity, though in most instances risk assessments were led and conducted by climate change officers. Climate change adaptation thus remained firmly rooted in each council’s environment or climate change team rather than being integrated more broadly into local planning and service management processes across councils. Even within the environment and climate change teams, the uptake of UKCP09 varied: some teams made regular use of them whilst others hardly used them at all. The use of climate projections thus appears not only to have been confined to certain (initial) stages of the adaptation planning process but also mostly to the respective officer or team tasked with the climate change agenda.

In terms of having something that is quite detailed and information heavy, I don’t think we’ve got an outlet for it…I would love to see it and look at the analysis of it and play around with it and see what happens, but in terms of usefulness outside of our team I just can’t see it because we have to be so simplified to people. (Employee of a Local Government, East Midlands England #3)

When the capacity to use climate projections is confined to very few people, competing pressures on said staff create a real risk of side-lining engagement with the projections. After the 2010 general election, local council efficiency savings of over 50 % (Hastings et al. 2015) and the dismantling of NI188 led LAs to redefine their priorities away from adaptation and towards mandatory frontline services and tasks (Fitzgerald and Lupton 2015). At the same time, staff redundancies of over 30 % (Hastings et al. 2015) in LAs or the transferral of existing staff to new roles led to a loss of expertise on climate projections.

And so we were progressing quite well, ‘til 2011, when all the indicators…went out the window with the new government, really. So it was all change again, and adaptation, at that point in particular, really dropped completely off the radar. (Employee of a Local Government, South East England #4)

The abolition of the indicator NI188 and the cuts to LA budgets happened at the same time, thus making it difficult to distinguish the exact cause of staffing losses. However, the interviewees considered that by making adaptation-related tasks voluntary, the abolition of the indicator NI188 put people focusing on those tasks at risk. Many, despite the varied criticisms of NI188, were thus sad to see it go.

Documentary analysis provides additional support for the lack of integration of climate projections into strategic and spatial planning in LAs. UKCP09 is not mentioned in any of the core strategies, and the two that refer to climate projections not only focus on headlines such as ‘summers are likely to be drier and hotter’ but in fact refer to climate predictions instead of climate projections. UKCP09 provides an array of possible future climate outcomes and their associated probabilities: mistaking them for predictions highlights lack of understanding of the nature and intended use of UKCP09 (Bray and von Storch 2009). Although adaptation plans and strategies refer to UKCP09 and climate projections more frequently, they remain focused on headlines or highlight temperature and precipitation changes without reflecting on how they might impact strategic and spatial planning.

In summary, there was initially a very ambitious approach to adaptation both nationally and locally on the basis of the regulatory framework around NI188. The demand for and use of climate projections in LAs emerged to respond to the requirements of NI188 and the push for UKCP09 by national departments and programmes. From 2010, the Conservative–Liberal coalition government introduced substantial changes to the regulatory and planning framework within which LAs are situated. Not only was the indicator set dismantled but the Localism Act 2011 promoted a voluntary approach to climate change adaptation that caused an ‘erosion of resolve’ within LAs to progress on adaptation (Dixon and Wilson 2013: 677). The Localism Act stipulates that local planning is to occur within the frame of a Local Plan that reflects the ‘local area’s vision’ (Parliament UK 2011). This arguably fails to sufficiently take into account impacts happening at higher scales (Wende et al. 2012). At even finer resolution, the government encourages the creation of community-led neighbourhood plans. These are not required to specifically consider sustainability or environmental issues as long as they align with the planning framework set out in the respective Local Plans. However, as 38 % of LAs do not have a Local Plan (TPI 2015), neighbourhood plans would be directly guided by the NPPF (Scott 2011), which itself has no specific stipulations for adaptation. Due to the changes imposed by the central government, adaptation is thus not sufficiently considered in local development planning (ASC 2012) and has been marginalised (Porter et al. 2015).

### Germany

In Germany, adaptation is considered a local matter. LAs have planning sovereignty despite having to conform to higher-level plans. Adaptation has been a voluntary task at local government level and doubts have been voiced whether any local action will be taken before adaptation becomes a mandatory task, especially in financially strained municipalities.

It is naturally always the case with voluntary tasks, that they always get put to the back of the queue. That is naturally the case with municipalities, and that is the majority in NRW, for example have financial problems, and then people like to or it is not otherwise possible, concentrate on things, that are legally mandated and as long as there is no legal mandate, to deal with the topic, many just simply ignore it. (Employee of a Local Government, NRW, Germany #1)

Although the Climate Protection Act in NRW sets out a roadmap for action on climate change, it is considered a political declaration of ‘advisory character’ due to the lack of clear targets on adaptation, responsibilities, and sanctions in the law. Whilst it sets out clear targets for mitigation, the article on adaptation leaves the extent of expected action on adaptation vague and unclear. As a result, there is not the kind of top-down guidance for progressing through the stages of local adaptation planning as there was in England under NI188.

Despite progress on adaptation at national level, adaptation at the local level still seems to be in the early stages. Climate projections are thus unlikely to play an important role in local decision-making processes in Germany. Our document review corroborates this: climate projections are only referred to in the climate change (adaptation) plans of three LAs and in the state adaptation plan. However, they are not mentioned in any of the local, regional or state-level planning documents in NRW. These findings indicate that, like in England, climate projections have not been integrated into local strategic and spatial planning.

On the other hand, we find that climate data, in the form of climate function maps and planning recommendation maps, have been widely used in the planning process for several decades in the larger LAs. Whilst climate change might be a more recent concern, the use of past and present climate data for the assessment of current vulnerabilities and exposure is well embedded in the German planning system. These maps are based on measured data of a variety of climate variables. Some LAs have even conducted consecutive analyses to establish the change in these variables. Planning maps indicate the present state of local climate and are subdivided into geographical areas with different microclimatic conditions and land-use characteristics (Heaphy 2014). This practice is guided by technical rules established by the Society of German Engineers (Matzarakis et al. 2008). The rules describe how the urban climate is to be represented and evaluated in maps that underpin urban and regional planning recommendations (Heaphy 2014). These maps often highlight potential heat islands and cold air paths and guide planners and developers on where additional development can or cannot take place. This planning style resonates with a vulnerability driven approach to adaptation (Adger 2006; Füssel 2007b), which prioritises current exposure and may thus see less need to use future climate projections. In fact, the current climate is considered by many LAs sufficient for planning purposes: ‘Yes well, I mean, in the present state of the climate, I can obviously already see a lot of mistakes, which will probably be the same with climate change’ *(Employee of a Regional Ministry, Germany #2)*. Climate change (adaptation) documents from a small number of the LAs consider analyses of current local climate a sufficient foundation for the development of an adaptation strategy. However, too narrow a focus on past and current vulnerability and exposure may not prepare German LAs sufficiently to cope with future climate change (Dilling et al. 2015).

The use of climate function and recommendation maps form an integral part of planning across LAs: ‘as an evaluation tool, it is a very important instrument here in the municipality. It is taken seriously’ *(Employee of a Local Government, NRW, Germany #3)*. Small-scale simulations are sometimes created with tools such as Envimet, a microclimate simulation tool, to establish how planning options would affect the local microclimate. That is, these tools are used to assess planning options and help with decision-making and resource allocation. These findings highlight that there is capacity, tools and a regulatory framework enabling the use of past and present climate data—but not projections of future climate—in local planning.

Some LAs have used climate projections to complement current climate maps to explore the future state of local climate. This demonstrates that climate projections can be used with well-established planning tools and highlights the potential capacity of the local planning system to extend its use of past and present climate data to include future climate projections. However, maps based on projections have often been used only internally, not for communication with elected council members or the public. The strictly regulated German planning system makes the use of climate projections in planning processes difficult because they do not fulfil the formal expectations about the nature of the information they provide (BMVBS 2013). Spatial planning recommendations have to be based on data that are spatially sufficiently concrete and accurate so that valid planning recommendations can be made (BMVBS 2013). This is something climate projections struggle to help with due to their inherent uncertainty. Not using climate projections is therefore less an issue of insufficient technical capacity or lack of tools but more an issue of lack of fit with regulatory and institutional requirements in the planning system and perceived communication and engagement challenges.

Finally, climate projections are not used simply because it is not required by the rules of federal and regional funding available to LAs for developing climate protection concepts. As many LAs have very constrained budgets, activities that are not mandatory are extremely unlikely to be undertaken.

The funding programme stipulates certain things, that one has to do and tick off the list, as otherwise one doesn’t get all of the funding. These climate projections were not specifically asked for…Only during the creation [of the climate protection concept] one becomes wiser, but then there simply wasn’t any time or budget left. (Employee of a Local Government, NRW, Germany #4).

However, making adaptation and its planning mandatory would be problematic as tight council budgets would not easily cope with additional expenses (Nalau et al. 2015). Whilst budgetary constraints in LAs are not unique to Germany, the particularities of the federal German funding system for climate protection plans state that statutory duties would not be fundable from national schemes (SUG 2013). Thus, making adaptation mandatory for LAs may mean foregoing potential sources of federal funding to help progress it.

### Comparative insights

In the preceding case studies, we explored the usability and adoption of climate projections within local adaptation planning in England and Germany. Whilst climate projections are not considered usable in local adaptation planning for a range of reasons in the two countries, we can nevertheless make a number of observations as to how the wider institutional context strongly influences the question of use and usability in both countries. Firstly, the lack of specific regulation on adaptation at the local government level results in little decision-making or actions taken that would require the use of climate information. With adaptation already several steps removed from the realities of fulfilling statutory requirements locally, using climate information to do so is even further away from current practice. Secondly, in both countries, planning law and the scales at which it is shaped plays a key role. By encouraging a more localised and less centrally regulated planning approach, adaptation in England, and with it the use of climate information, has been subsumed by more pressing local demands. Germany, on the other hand, with its strict and clearly set out national and regional planning laws, does not allow local decision-makers, even if strong buy-in into adaptation is present in certain LAs, to incorporate uncertain climate information as part of the planning process. Lastly, both case studies highlight the strong influence of the provision (or lack thereof) of financial resources and capacity. Without these, investing in adaptation decisions and actions, and using climate information to help do so, is currently seen as an unaffordable luxury.

These comparative insights show that just as the progress on adaptation at the local scale can be helped or hindered by the wider rules, policies and regulations, so can the usability of climate projections. Our findings are largely based on interviews within our three focus regions and are thus spatially limited. The findings also reflect a snapshot in time. Nevertheless, our additional interviews from outside the focus regions, whilst limited in number, support our findings. They show that our findings are not due to regional particularities but instead highlight that LAs in both countries are equally subject to the external influence of the national planning frameworks, laws and regulations.

If the wider setting, however, proves not to be conducive to the use of climate projections for adaptation planning, we need to ask ourselves whether our endeavours to increase the usability and adoption of climate projections are futile. The English experience raises the question to what extent the discussion on the usability of climate projections at a local level is sensible at the moment. It rather looks as if the discussion should be about the creation of a new external institutional setting which would be conducive to fostering local adaptation planning, with or without the use of climate projections. A shift in attention is also necessary in Germany, where the lack of fit is more likely to be addressed effectively if planning regulations become more amenable to using climate projections as data for evidence-based decision-making. Whilst striving to ensure greater usability at local level, we cannot let our attention slip away from the question as to how we create a wider setting that encourages both local adaptation planning and the use of climate projections at the same time.

## Conclusion

By using two case studies, Germany and England, we have shown in this article that conceptual developments in the literature on climate information usability (Lemos et al. 2012) could benefit substantially from drawing more explicitly on the well-researched factors influencing adaptation planning. Addressing the question of usability is not just about better understanding the interplay between what science can provide and what users need or want, but also about what users can actually *do* within the political and economic constraints within which they act. A more nuanced understanding of the ‘what can be done’ can be achieved by looking beyond the immediate institutional context within which users and producers interact and looking outwards to the wider setting and legal and regulatory system within which they are placed. The developments and changes in the wider setting may in turn be better understood through insights from policy studies on such questions as policy innovation and adaptation (Massey and Huitema 2016) as well as on the impact of policy dismantling (Jordan et al. 2013).

Adaptation has long been considered highly contextual (Füssel 2007a) and so is usability of climate data and projections. We may run the risk of over-focusing on a usability concept that is too narrowly defined and continue to put forward ever smarter solutions through tailoring information, whilst being ignorant of the wider context, which in turn impacts the usability of such solutions. This is not to say that we do not need to continue to gain a better understanding of the user–producer interface in order to make information more usable (Lemos et al. 2012). Rather, it is to say that we also need a better understanding as to how to nest the usability debate within the bigger institutional and contextual debate of adaptation planning.

## Electronic supplementary material

Below is the link to the electronic supplementary material.

Focus area description (DOCX 17 kb)

Overview of planning and climate change (adaptation documents reviewed) (DOCX 14 kb)
