# Supplementary material for: Adaptation planning and the use of climate change projections in local government in England and Germany
Source: Reg Environ Change. 2016 Jul 28;17(2):425–35. doi: 10.1007/s10113-016-1030-3 (PMC7114967; doi:10.1007/s10113-016-1030-3)
Supplement: Supplementary file 1 — Focus area description (DOCX 17 kb) [file 10113_2016_1030_MOESM1_ESM.docx]

# Journal name: Regional Environmental Change

**Article title:** Adaptation planning and the use of climate change projections in Local Government in England and Germany

**Authors:** Lorenz, S., S. Dessai, P. M. Forster and J. Paavola

**Affiliation:** School of Earth and Environment and Centre for Climate Change Economics and Policy, University of Leeds, Leeds, LS2 9JT, UK, ORCID ID orcid.org/0000-0002-9124-9690

**Author for correspondence:** [S.Lorenz@leeds.ac.uk](mailto:S.Lorenz@leeds.ac.uk)

# Supplementary material 1

**Focus area description**

Our empirical data collection focused on the South East Region and the East Midlands Region of England, as they encompass a range of climate change impacts demanding adaptation whilst showcasing socio-economic and demographic diversity. The South East is the country’s most populous region with ~8.7 million inhabitants (ONS 2014a), 75% of which live in urban areas (Causer and Park 2011). It is second only to London, in terms of economic performance, contributing almost 15% to the UK’s gross value added (GVA) (ONS 2014b). The South East is impacted by flooding with 25% of properties at risk, but after London, the region is also likely to suffer the most from extreme heat events (Climate UK 2012a), especially because of its higher proportion of older people (Causer and Park 2011).

The East Midlands Region currently has 4.6 million residents (ONS 2014a), but it is expected to see the highest population growth amongst the English regions over the next two decades (Beaumont 2009). The region contributes almost 6% of UK GVA (ONS 2014b). The regional economy was originally based on the textile and coal industry and manufacturing together with agriculture are still drivers of the economy (Beaumont 2009). Flooding especially at the coast but also water shortages for agricultural production are key projected impacts from climate change (Climate UK 2012b).

In Germany, our study focused on the state of North Rhine-Westphalia (NRW). It is the industrial heartland of the country as well as a state in which adaptation policy is being increasingly legislated. NRW is Germany’s most populous state with ~17.6 million inhabitants (SB 2013). The state contributes almost 22% to German GVA (SB 2014), with the financial, insurance and business sectors dominating. The industrialised zone in the Rhine Valley is considered as one of Germany’s most sensitive regions to a number of climate change impacts (Rannow et al. 2010), with flooding and heat stress projected to be causing the largest impacts (Rannow et al. 2010, Schröter et al. 2005).

**References**

Beaumont J (2009) Portrait of the East Midlands. Office for National Statistics. <http://tinyurl.com/pwuzznf>. Accessed 5 July 2015

Causer P, Park N (2011) Portrait of the South East. Office for National Statistics. <http://tinyurl.com/nkl4doo>. Accessed 5 July 2015

Climate UK (2012a) A Summary of Climate Change Risks for South East England. Climate South East. <http://tinyurl.com/ocy64nr>. Accessed 5 July 2015

Climate UK (2012b) A Summary of Climate Change Risks for the East Midlands. Climate East Midlands. <http://tinyurl.com/qxq8y4x>. Accessed 5 July 2015

Office for National Statistics (ONS) (2014a) 2012-based Subnational Population Projections for England. Office for National Statistics. <http://www.ons.gov.uk/ons/rel/snpp/sub-national-population-projections/2012-based-projections/stb-2012-based-snpp.html>. Accessed 5 July 2015

Office for National Statistics (ONS) (2014b) Regional Gross Value Added (Income Approach), December 2013. Office for National Statistics. <http://www.ons.gov.uk/ons/rel/regional-accounts/regional-gross-value-added--income-approach-/december-2013/stb-regional-gva-2012.html>. Accessed 5 July 2015

Rannow S, Loibl W, Greiving S, Gruehn D, Meyer BC (2010) Potential impacts of climate change in Germany-Identifying regional priorities for adaptation activities in spatial planning. Landscape and Urban Planning 98: 160-171. doi: 10.1016/j.landurbplan.2010.08.017

Schröter D, Zebisch M, Grothmann T (2005) Climate change in Germany-vulnerability and adaptation of climate-sensitive sectors. Deutscher Wetter Dienst. <http://tinyurl.com/og8zbkw>. Accessed 5 July 2015

Statistisches Bundesamt (SB) (2013) Gebiet und Bevölkerung – Fläche und Bevölkerung. Statistisches Bundesamt. <http://www.statistik-portal.de/Statistik-Portal/de_jb01_jahrtab1.asp> Accessed 22 May 2015

Statistisches Bundesamt (SB) (2014) Volkswirtschaftliche Gesamtrechnungen – Bruttowertschöpfung. Statistisches Bundesamt. <http://www.statistik-portal.de/statistik-portal/de_jb27_jahrtab66.asp>. Accessed 22 May 2015
