# Supplementary material for: Adaptation planning and the use of climate change projections in local government in England and Germany
Source: Reg Environ Change. 2016 Jul 28;17(2):425–35. doi: 10.1007/s10113-016-1030-3 (PMC7114967; doi:10.1007/s10113-016-1030-3)
Supplement: Supplementary file 2 — Overview of planning and climate change (adaptation documents reviewed) (DOCX 14 kb) [file 10113_2016_1030_MOESM2_ESM.docx]

# Journal name: Regional Environmental Change

**Article title:** Adaptation planning and the use of climate change projections in Local Government in England and Germany

**Authors:** Lorenz, S., S. Dessai, P. M. Forster and J. Paavola

**Affiliation:** School of Earth and Environment and Centre for Climate Change Economics and Policy, University of Leeds, Leeds, LS2 9JT, UK, ORCID ID orcid.org/0000-0002-9124-9690

**Author for correspondence:** [S.Lorenz@leeds.ac.uk](mailto:S.Lorenz@leeds.ac.uk)

# Supplementary material 2

We analysed climate change (n = 6) and climate change adaptation strategies and plans (n = 4) for 8 out of 14 LAs in the two English regions under study. Only two LAs had both types of strategies, and six LAs did not have either publicly available. As 10 of the 14 LAs are local planning authorities, we also reviewed their core strategies, which determine the overarching guidance for local planning. In Germany, we reviewed the NRW state development plan (*Landesentwicklungsplan*), the regional plans for the districts in NRW (*Regionalplan*) (n = 14) and the publicly available local land utilisation plans (*Flächennutzungsplan*) (n = 6) for those LAs in NRW we conducted interviews in. In addition, we examined the climate protection (and adaptation) concepts and plans, which were publicly available for 10 out of the 15 LAs in NRW we interviewed in (n = 9, as two of the LAs commissioned a joint concept). The concepts mainly focused on mitigation and were funded either nationally or by the state environment ministry. However, ‘special concepts’ that focus on adaptation and integrated concepts looking at both mitigation and adaptation are also supported.
